# Integrated metabolic and genetic analysis reveals distinct features of human differentiated thyroid cancer

**DOI:** 10.1002/ctm2.1298

**Published:** 2023-06-14

**Authors:** Eduardo Cararo Lopes, Akshada Sawant, Dirk Moore, Hua Ke, Fuqian Shi, Saurabh Laddha, Ying Chen, Anchal Sharma, Jake Naumann, Jessie Yanxiang Guo, Maria Gomez, Maria Ibrahim, Tracey L. Smith, Gregory M. Riedlinger, Edmund C. Lattime, Stanley Trooskin, Shridar Ganesan, Xiaoyang Su, Renata Pasqualini, Wadih Arap, Subhajyoti De, Chang S. Chan, Eileen White

**Affiliations:** ^1^ Rutgers Cancer Institute of New Jersey New Brunswick New Jersey USA; ^2^ Department of Molecular Biology and Biochemistry Rutgers University Piscataway New Jersey USA; ^3^ Department of Biostatistics and Epidemiology Rutgers School of Public Health Piscataway New Jersey USA; ^4^ Department of Medicine Robert Wood Johnson Medical School Rutgers University New Brunswick New Jersey USA; ^5^ Department of Chemical Biology Rutgers Ernest Mario School of Pharmacy Piscataway New Jersey USA; ^6^ Rutgers Cancer Institute of New Jersey Newark New Jersey USA; ^7^ Division of Cancer Biology Department of Radiation Oncology Rutgers New Jersey Medical School Newark New Jersey USA; ^8^ Department of Surgery, Robert Wood Johnson Medical School Rutgers University New Brunswick New Jersey USA; ^9^ Division of Hematology/Oncology Department of Medicine Rutgers New Jersey Medical School Newark New Jersey USA; ^10^ Ludwig Princeton Branch, Ludwig Institute for Cancer Research Princeton University Princeton New Jersey USA

**Keywords:** intra‐tumour heterogeneity, metabolism, metastases, thyroid cancer

## Abstract

**Background:**

Differentiated thyroid cancer (DTC) affects thousands of lives worldwide each year. Typically, DTC is a treatable disease with a good prognosis. Yet, some patients are subjected to partial or total thyroidectomy and radioiodine therapy to prevent local disease recurrence and metastasis. Unfortunately, thyroidectomy and/or radioiodine therapy often worsen(s) quality of life and might be unnecessary in indolent DTC cases. On the other hand, the lack of biomarkers indicating a potential metastatic thyroid cancer imposes an additional challenge to managing and treating patients with this disease.

**Aim:**

The presented clinical setting highlights the unmet need for a precise molecular diagnosis of DTC and potential metastatic disease, which should dictate appropriate therapy.

**Materials and methods:**

In this article, we present a differential multi‐omics model approach, including metabolomics, genomics, and bioinformatic models, to distinguish normal glands from thyroid tumours. Additionally, we are proposing biomarkers that could indicate potential metastatic diseases in papillary thyroid cancer (PTC), a sub‐class of DTC.

**Results:**

Normal and tumour thyroid tissue from DTC patients had a distinct yet well‐defined metabolic profile with high levels of anabolic metabolites and/or other metabolites associated with the energy maintenance of tumour cells. The consistency of the DTC metabolic profile allowed us to build a bioinformatic classification model capable of clearly distinguishing normal from tumor thyroid tissues, which might help diagnose thyroid cancer. Moreover, based on PTC patient samples, our data suggest that elevated nuclear and mitochondrial DNA mutational burden, intra‐tumour heterogeneity, shortened telomere length, and altered metabolic profile reflect the potential for metastatic disease.

**Discussion:**

Altogether, this work indicates that a differential and integrated multi‐omics approach might improve DTC management, perhaps preventing unnecessary thyroid gland removal and/or radioiodine therapy.

**Conclusions:**

Well‐designed, prospective translational clinical trials will ultimately show the value of this integrated multi‐omics approach and early diagnosis of DTC and potential metastatic PTC.

## Graphical abstract

- Differentiated thyroid cancer presents a well‐defined metabolic profile that can be used for diagnosis and management.
- Integrated metabolomics, genomics and cytopathologic analysis might indicate the metastatic potential of papillary thyroid cancer.
- Papillary thyroid cancer presents high metabolic, transcriptional and immunogenic, but not genetic, intra‐tumour heterogeneity.

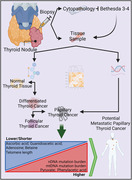

## 1 INTRODUCTION

The Surveillance, Epidemiology and End Results (SEER) database estimated that thyroid cancer represents ∼2.2% of all new cancer cases in the United States in 2023 and is responsible for ∼2120 deaths in the same period. Follicular thyroid and parafollicular C cells are the two endocrine cells from which thyroid cancers putatively originate. However, thyroid tumours with the largest worldwide incidence arise from follicular cells. They are generically classified as undifferentiated thyroid cancer (anaplastic) and differentiated thyroid cancer (DTC). DTC is divided into three histopathologic categories, namely papillary thyroid cancer (PTC), follicular thyroid cancer (FTC), and Hurthle cell carcinoma (a rare variation of FTC).^1^, ^2^

Currently, one concern related to DTC is its unexplained increase in the past 40 years. Although the incidence of DTC has been growing, the mortality of DTC patients has paradoxically remained stable.^3^, ^4^ Some investigators suggested that overdiagnosis (caused by the increased ability to detect indolent nodules and papillary cancers that would never cause symptoms or be life‐threatening) may play a role.^5^, ^6^ However, the increased incidence of nodules larger than 5 cm is a genuine concern,^7^ as they are generally symptomatic and must be treated by surgical removal of the thyroid gland and, if cancer is present, both by adjuvant radioiodine therapy in certain cases.^8^, ^9^ Moreover, the epidemiologic data suggest that both the overdiagnosis and truly increased incidence of clinical thyroid nodules occur concomitantly, suggesting that a more accurate methodology for identifying tumours of clinical concern is desirable in this setting.

Finally, an increasing number of misdiagnosed thyroid cancer patients undergo partial or complete surgical removal of their thyroid gland,^8^ leading to several problems, including inappropriate/unnecessary surgical or radioiodine therapy.^9^, ^10^, ^11^

Therefore, the diagnostic method for differentiating benign from malignant tumours and indolent versus aggressive thyroid cancers must be improved.^12^ The current gold standard procedure to diagnose thyroid cancer is still the cytopathologic analysis of thyroid nodule samples obtained from percutaneous fine‐needle aspiration (FNA) by using the Bethesda Classification System.^8^, ^13^, ^14^ Unfortunately, FNA with pathologic analysis is inconclusive in up to ∼25% of cases, bringing into question the choice of therapy.^10^, ^15^, ^16^ In order to overcome this challenge, molecular testing for the precise diagnosis of indeterminate thyroid nodules was developed. The most used are RNA expression–based detection (Veracyte^17^) or identification of somatic mutations (ThyroSeq^18^). Nevertheless, both methods show their limitations^19^ which might be complemented by new methods of diagnosis, such as metabolic profiling.

Moreover, a small percentage of these tumours belong to high‐risk thyroid cancer variants that often generate distant metastasis, with a very poor prognosis compared with non‐metastatic thyroid cancer^20^; in addition, up to 20% of patients with DTC may have residual tumours left behind after surgical or radioactive iodine treatment.^1^, ^21^, ^22^, ^23^, ^24^ Therefore, there is a clear need for improved procedures and biomarkers to distinguish between benign nodules and low‐risk and high‐risk thyroid cancers.

Here we evaluate the metabolomic profile of normal gland and thyroid tumours from DTC patients (*n* = 20) with different features (e.g. sex, stage and metastatic status), showing that the metabolic profile of DTC tumour cells remains consistent and well defined and may potentially be used to distinguish normal thyroid gland tissue from cancer. Matched tissues from these patients (*n* = 10), including normal and tumour samples, were subjected to whole‐genome sequencing (WGS). Unexpectedly, the WGS results indicated that other features beyond the mutational profile, such as mitochondrial and nuclear DNA (mtDNA and nDNA) mutational burden and telomere length, may also serve as candidates of biomarkers for metastatic PTC, including local lymph node metastasis (LM) and high‐risk PTC presenting with distant metastases.

We also compared the intra‐tumour heterogeneity (ITH) among multiple samples from the same thyroid tumour. We observed that histopathological heterogeneity strongly correlates with post‐transcriptional events (such as gene expression and metabolic rewiring) independently of the conserved mutational profile. Together, these results highlight the translational importance of integrated omics for thyroid cancer diagnosis, prognosis and management.

## 2 RESULTS

### 2.1 Differentiated thyroid cancer has a well‐defined metabolic profile

**FIGURE 1:**
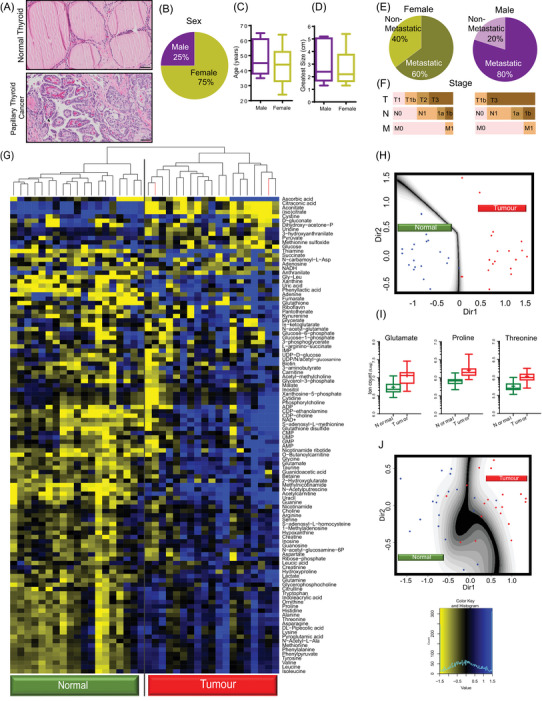
Differentiated thyroid cancer (DTC) presents a well‐defined metabolic profile. (A) H&E scanned slides of normal thyroid tissue and papillary thyroid cancer (scale bar 50 μm); profile of DTC patients considering (B) sex, (C) age and (D) the greatest size of the primary DTC tumour; (E) thyroid cancer risk, metastatic (local and distant metastasis) and non‐metastatic, per sex; (F) classification of thyroid cancer according to histopathologic analysis in surgical report; primary tumour (T), local metastasis (lymph nodes [N]) and distant metastasis (other organs [M]); (G) heat map of metabolites extracted from primary thyroid cancer and normal thyroid tissue. The follicular cancer samples are pointed red in the clustering tree; (H) mclust analysis considering the six most significant altered metabolites. All the points except one lie on their respective sides. The one exception lies within the boundary error region; (I) box plot of the three metabolites with lower *p*‐value used to perform mclust analysis (J) with the lower number of biomarkers possible.

In order to evaluate the metabolomic profile of DTC, we used normal tissue and primary thyroid tumour obtained from partial or total surgical thyroid resections (Figure 1A). The histologic subtype was classified, and only the patients with DTC were selected. This cohort comprised 20 patients; a total of 18 DTC patients (95%) had their tumours classified as PTC, whereas the other two had FTC (Table S1). Most of the patients were female (75%) and, on average, were slightly younger than the men (Figure 1B,C). Although the average size of the primary tumour in both sexes was similar and not significantly different, in this series, the percentage of metastatic DTC samples (including distant and local metastasis) was higher in men, which may suggest a more aggressive disease, late clinical diagnosis or both (Figure 1D–F). The cohort of patients evaluated here is generally representative of the natural history of DTC.

**TRANSLATIONAL RELEVANCE**

In this article, we propose new integrated metabolic, genomic and cytopathologic methods to diagnose differentiated thyroid cancer when the conventional methods fail. Moreover, we suggest metabolic and genomic markers to help predict high‐risk papillary thyroid cancer. Both may be important tools to avoid unnecessary surgery and/or radioiodine therapy that can worsen the quality of life for patients more than living with an indolent thyroid nodule.

**STUDY APPROVAL**

All patients consented to submit their samples to this protocol in writing. All tissue samples were acquired by partial or total thyroid dissection according to the approved protocol CINJ 001724 – Pro20170001082 and Institutional Review Board (IRB) approval.

One of our goals was to identify metabolic biomarkers predictive of whether or not a tissue sample is normal or malignant thyroid tissue. We also evaluated whether the metabolic changes might shed light on the molecular mechanisms of metastatic DTC. We focused on polar metabolites (*n* = 110) from each sample (see Supplementary Results – Metabolomics). These analyses clearly resulted in a DTC metabolic profile distinct from normal thyroid (Figure 1G). Next, we aimed to identify an informative subset of biomarkers, which may effectively differentiate the two types of tissue (normal and cancer) based on their metabolic profile. As a first step, we constructed a predictive model (Normal vs. Tumour), which used ordinary logistic regression to assess the relationship of each compound to the sample type. We selected the metabolic biomarkers with *p*‐values ≤.20/110 = .0018, thereby practically reducing the number of candidate biomarkers (from 110 to 19). We next used cross‐validated Lasso logistic regression by using the ‘optL1’ function in the R package ‘penalized’ to find the penalty parameter that minimizes the 10‐fold cross‐validated penalized likelihood.^25^ We then used the ‘penalized’ R function in this package with this penalty parameter to identify the optimal subset of metabolic biomarkers (*n* = 6) that were most effective in predicting normal tissue versus cancer. The six biomarkers found were aconitate, glycine, inosine, isoleucine, proline and taurine (Figure S1A). These results clearly show that applying the aforementioned predictive model in the metabolomic analysis distinguishes normal thyroid tissue from primary thyroid tumours.

To illustrate the ability to use these six compounds to separate normal and cancer samples, we next used a procedure known as ‘mclust’ that identifies the projection of the six‐dimensional predictors onto two dimensions that optimally separate the two groups^26^, ^27^ (Figure 1H). These results suggest that the six predictors selected may differentiate normal biopsy samples from thyroid tumours. To better evaluate the classification error, we applied the 10‐fold cross‐validation methods, which yielded an error rate of .1316 and a standard error of .0451, clearly differentiating this error rate from the null hypothesis value of .5 (Figure S1B).

Moreover, we considered whether an even smaller subset of biomarkers could be suitable for adequate classification. To this end, we performed the same procedure with five, four, or three of the most effective metabolic biomarkers in predicting the phenotype. However, the model with fewer predictors had lower power and misclassified many samples (Figure S1C,D), but in all cases, the error rates are below .5. For instance, selecting the three biomarkers (i.e. glutamate, proline and threonine) (Figure 1I) generated three misclassifications per sample type, all lying in the error boundary (Figure 1J). Applying 10‐fold cross‐validation, we could rely on an error rate of .21 and a standard error of .051, suggesting that this more straightforward procedure could be helpful in the absence of a more extensive metabolic panel. For instance, all proteogenic amino acids are present at higher levels in tumour samples relative to controls (Figure S1E). However, applying other metabolites different than the most altered ones shown here must still be carefully validated in future studies. Thus, our data suggest that a histopathologic analysis supplemented by a small validated metabolomic biomarker panel might improve DTC diagnosis.

### 2.2 The metabolic alterations of DTC are enriched in energy maintenance and anabolic metabolism

**FIGURE 2:**
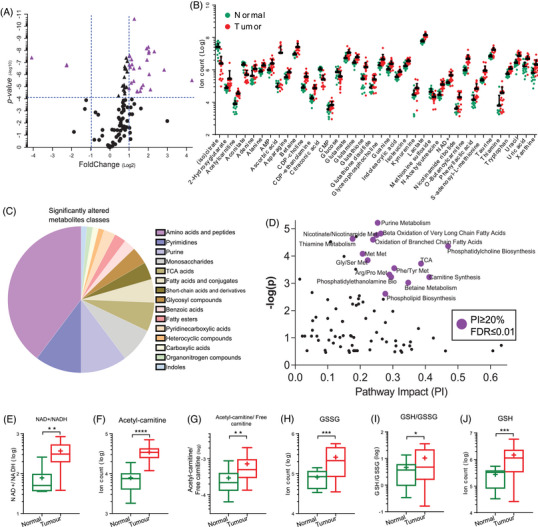
Differentiated thyroid cancer (DTC) prioritized metabolism of building blocks and energy maintenance: (A) volcano plot of metabolites extracted from DTC patients. The purple triangles highlight the significantly altered metabolites (*p‐*value ≤.01/110) with fold change higher than double or less than a half comparing cancer versus normal thyroid tissues. The levels of these metabolites are graphically expressed in (B); data presented as mean (SEM); (C) the most altered classes of metabolites in DTC. (D) Pathways impact analysis. Purple circles show the metabolic pathways impacted in more than 20%, with a false discovered ratio equal to or lower than .01. Box plot of metabolites involved in energy maintenance (E–G) and oxidative stress (H–J). *t*‐Test **p ≤ .01, ***p ≤ .001 and ****p ≤ .0001.

A volcano plot showed that most metabolites are present at higher levels in DTC (Figure 2A). As 45 of 110 metabolites (41%) were significantly altered (adjusted *p*‐value ≤.01/110), we applied a more restrictive analysis considering only the metabolites that have fold change more than double, or less than half, in normal thyroid versus cancer tissue (Figure 2A,B). Thus, we found the most affected metabolite classes and the pathways these biochemical alterations impact.

According to this stringent criterion, ∼70% of the metabolites are amino acids, purines, pyrimidines, 1‐carbon metabolism intermediates, tricarboxylic acid (TCA) cycle components and fatty acids (Figure 2C). The most altered metabolites in malignant DTC are involved in two fundamental processes: metabolism of building blocks and energy maintenance. Indeed, the pathway impact analysis revealed that the main altered pathways in DTC are the TCA cycle, beta‐oxidation of fatty acids, amino acids and purine/pyrimidine metabolism (Figure 2D). A high‐energy requirement of thyroid tumour cells might be responsible for the altered TCA intermediate metabolites, as suggested by the higher NAD+/NADH ratio in tumours (Figure 2E). However, the most robust result linked to energy maintenance was the altered levels of several metabolites from lipid metabolism. For instance, a lower level of free carnitine in tumour cells may be one example because one of its main metabolic roles is to shuttle long‐chain fatty acids across the mitochondrial membrane to be burned by β‐oxidation (Figure 2G). Furthermore, the higher acetyl‐carnitine/free carnitine ratio also suggests that DTC might have altered β‐oxidation activity, consistent with previous observations.^28^

As with other malignant tumour cells, thyroid cancer cells are driven to increase the proliferative ratios, requiring anabolism to produce building blocks. Moreover, rapid proliferative growth caused by oncogenic activity might generate harmful oxidative species.^29^ These results may perhaps explain the altered levels of intermediates of 1‐carbon metabolism, *S*‐adenosyl methionine cycle and glutathione (GSH) (Figure 2B,H–J).

### 2.3 A panel of six metabolites is associated with metastatic papillary thyroid cancer

DTC with lymph node involvement has a better prognostic than patients with distant metastatic disease. However, patients with local lymph node metastases are the ones more likely to develop distant metastases. The percentage of so‐called metastatic (including local and distant) DTC reported in the literature is relatively low (∼5%), but their prognosis is much poorer than those patients with non‐metastatic DTC. Together, these data reinforce the unmet need for early diagnosis of tumours with potentially aggressive metastatic behaviour.^20^, ^23^

**FIGURE 3:**
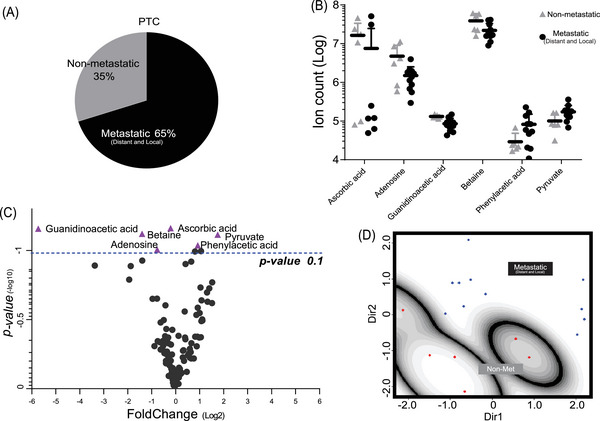
Six metabolites can help to identify metastatic papillary thyroid cancer (PTC): (A) distribution of non‐metastatic and metastatic (local and distant) PTC patients; (B and C) most altered metabolites in metastatic PTC (*p*‐value ≤.1) as follow: ascorbic acid (*p =* .06), guanidinoacetic acid (*p =* .06), betaine (*p =* .07), pyruvate (*p =* .07), phenylacetic acid (*p =* .09) and adenosine (*p =* .09); (D) mclust projection showing the isolation of metastatic samples in a single group.

Most cases submitted to thyroid dissection in our cohort were metastatic (Figure 3A). As there is a great need for reliable biomarkers for the metastatic potential of this tumour subset,^30^ we used such cohort composition in our favour to identify specific biomarkers that could help determine if a primary tumour was likely to metastasize to local lymph nodes or distant organs. We purposely removed the two samples from this analysis classified as FTC to avoid any noise in the classification. Therefore, we analysed non‐metastatic (*n* = 6) and metastatic (*n* = 11) PTC patient samples.

The ordinary logistic regression model was applied, evidencing six biomarkers with a *p*‐value lower than .1: adenosine, ascorbic acid, betaine, guanidoacetic acid, phenylacetic acid and pyruvate (Figure 3B,C). None of the metabolites was found below the level of the Bonferroni‐based cut‐off (adjusted *p*‐value ≤.0004545); however, we must consider this an extremely stringent criterion because of the much higher number of biomarkers compared with the number of samples. Nevertheless, the positive coefficients indicated by a non‐adjusted *p*‐value (*p*‐value ≤.1) presented a positive correlation between these six metabolite levels and metastatic PTC tumours.

These six metabolites were considered for mclust analysis followed by 10‐fold cross‐validation (Figure 3D). All metastatic PTC patients were classified as a single group (error rate .1176 with SE = .0667). Notably, the 95% confidence interval excludes .5, indicating that this classification is statistically significant, and that the abovementioned procedure is robust enough to distinguish non‐metastatic from metastatic PTC samples in this set.

### 2.4 High nuclear and mitochondrial DNA mutation burden and shorter telomere length correlate to metastatic PTC

**FIGURE 4:**
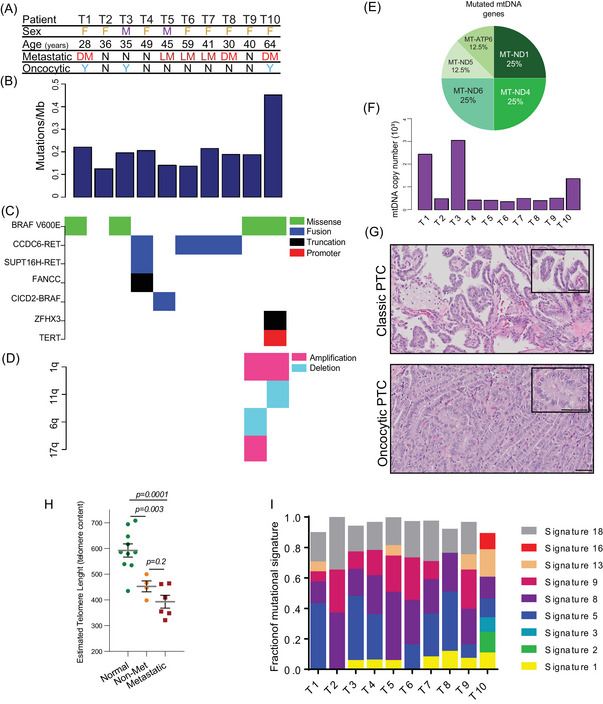
Whole‐genome sequencing of primary cancers suggests genetic features associated with metastatic papillary thyroid cancer (PTC): (A) general information and tumour characterization per patient across the cohort. Female and male (F and M); normal and non‐oncocytic samples (N); distant metastasis (DM), which includes other organs different than local lymph nodes and thyroid (please check patient information in Table S1 for more details); local metastasis (LM) in head neck lymph nodes; (B) histogram presenting nuclear DNA mutation burden per sample; (C) driver mutations in PTC; (D) chromosome alterations; (E) distribution of mtDNA mutations found in the genes that encode proteins of the mitochondrial electron transport chain; (F) histogram presenting mtDNA copy number per sample; (G) H&E of scanned slides showing the histology of PTC classic type and PTC with oncocytic alterations (scale bar 50 μm); (H) estimated telomere length in normal (*n* = 10), non‐metastatic (*n* = 4) and metastatic PTC (*n* = 6), data presented as mean (SEM) one‐way ANOVA, corrected Benjamini Yekutieli; (I) COSMIC mutational signatures in PTC.

Ten PTC tumours with matched normal samples were randomly selected and submitted to WGS with an average depth of 60× coverage. Four of these tumours were non‐metastatic, and six were metastatic (local and distant metastasis) (Figure 4A). The overall nDNA mutation burden in PTC was low, generally less than .2 mutations per Mb (Figure 4B). However, five PTC samples presented an nDNA mutation burden above this threshold. Four of them are metastatic, although not statically significant (*p =* .2). This result might perhaps suggest a possible link between high nDNA mutation burden and metastatic potential in PTC (Figure S2A), a lead to be further verified in future studies. We identified 7 driver mutations in this set of 10 PTC samples (Figure 4C). Five of these tumours had a BRAF mutation, four had the hotspot V600E mutation and one had a CICD2‐BRAF fusion. The structural variant (SV) analysis identified the other four thyroid tumours with inversions that resulted in CCDC6‐RET fusions. Although the breakpoints of the four inversions differed, they all occurred within intron‐1 of CCDC6 and intron‐11 of RET, resulting in the same fusion protein. Interestingly, our classification model based on the metabolic profile could not distinguish between samples with BRAF or RET alterations (Figure S2B,C). Although BRAF^V600E^ presented some association with more aggressive PTC elsewhere,^31^, ^32^ we could not find any correlation between this mutation and gain of aggressiveness in our cohort, as also reported by other groups.^33^

Only two tumours showed meaningful copy number alterations. The tumour sample T9 had chromosome 1q duplication, 16q deletion and 17q duplication (Figure 4D). The other tumour sample (T10) had chromosome 1q duplication and 11q deletion.

The WGS average depth of coverage allows us to determine the mtDNA mutations by using GATK and Mutect2 (see Section 4) and found missense and truncated mitochondrial gene mutations in six PTC samples (Table S2). Most mtDNA mutations were found in genes that encode proteins from the electron transport chain complex I, mainly in MT‐ND1, MT‐ND4 and MT‐ND6; these alterations were also reported in previous articles (Figure 4E).^34^, ^35^ Metastatic PTC samples had a higher allelic frequency of mtDNA mutations (Figure S2D), suggesting that a high mtDNA mutation burden might be associated with metastatic risk.

The histopathology analysis of the samples with higher mtDNA copy numbers (namely T1, T3 and T10) yielded PTC with oncocytic features (Figure 4F,G), with a ubiquitous BRAF^V600E^ mutation. Interestingly, one of the tumour samples (T10) had several mutations in mtDNA: MT‐ND1, MT‐ND4 and MT‐ATP6. The mutation in MT‐ND1 has an allele frequency of .83, demonstrating that this mutation is homoplastic, a feature found only in this tumour sample.

We also estimated the telomere length of all samples, including the normal thyroid tissue (Figure 4H). The estimated telomere length of normal tissue compared with the tumour samples, metastatic or not, is much longer. Nonetheless, a clear trend indicates that metastatic PTC has a shorter telomere length than non‐metastatic tumours.

Despite the absence of a specific gene mutation that could predict a more aggressive PTC, these results suggest that some genetic features, such as high nDNA and mtDNA mutation burden and shorter telomere length, might be associated with metastatic PTC phenotype.

### 2.5 Papillary thyroid cancer mutational signatures

The genetic sequencing of PTC samples presented nine different mutational signatures among the patients (Figure 4I). The most common mutational signature is #8, which was present in all samples. This signature is associated with possible radiation exposure, one of the leading causes of thyroid cancer.^36^, ^37^ The second most common mutational signature was #18, which was found in nine patients. Curiously, signature #18 is linked to colibactin exposure. Colibactin is a mutagenic agent secreted by *Escherichia coli*, which is highly associated with genotoxic colon cancer signatures but rarely reported in thyroid cancer.^38^ Additionally, the enrichment of signatures #8, #9 and #18 correlate with a substantial number of C > A mutations in this cohort, a pattern of mutations associated with damage caused by reactive oxygen species.^39^

The mutational signatures #1 and #5 were found in most malignant tumour samples. These signatures correlate with the age at the time of cancer diagnosis and hence the nDNA mutation burden. However, in this cohort, the correlation between signatures #1 or #5 with nDNA mutation burden or age was not observed (Figure S2E,F).

The mutational signatures and the mutated gene pattern did not distinguish aggressive PTC. Nonetheless, one patient sample (T10) presented the most advanced diseases with local and distant metastases. This patient also has a distinct characteristic presenting seven of nine (78%) listed signatures (Figure 4I). This is the only sample demonstrating mutational signatures linked to age, smoking and high numbers of insertion–deletion mutations (signatures #2, #3 and #16). All these features were present in this sample, which belonged to the oldest patient, who was a former smoker and whose sample presented an increased number of insertions‐deletions (indels) and had the highest nDNA mutation burden (Figure 4B,D and Table S1). Moreover, the tumour sample T10 was the only sample that presented three potential driver mutations: BRAF, ZFHX3 and telomerase reverse transcriptase (TERT) promoter (Figure 4C). This sample had a high mtDNA burden, and it was the only sample with a homoplastic mutation (in MT‐ND1 gene). Furthermore, the T10 patient also presented multifocal nodules and oncocytic sites in both thyroid glands. All these features suggest a history of high ITH that might perhaps play a role in high‐risk PTC and deserves to be studied.

### 2.6 Papillary thyroid cancer tumours present high metabolic, transcriptional and immunogenic, but not genetic ITH

**FIGURE 5:**
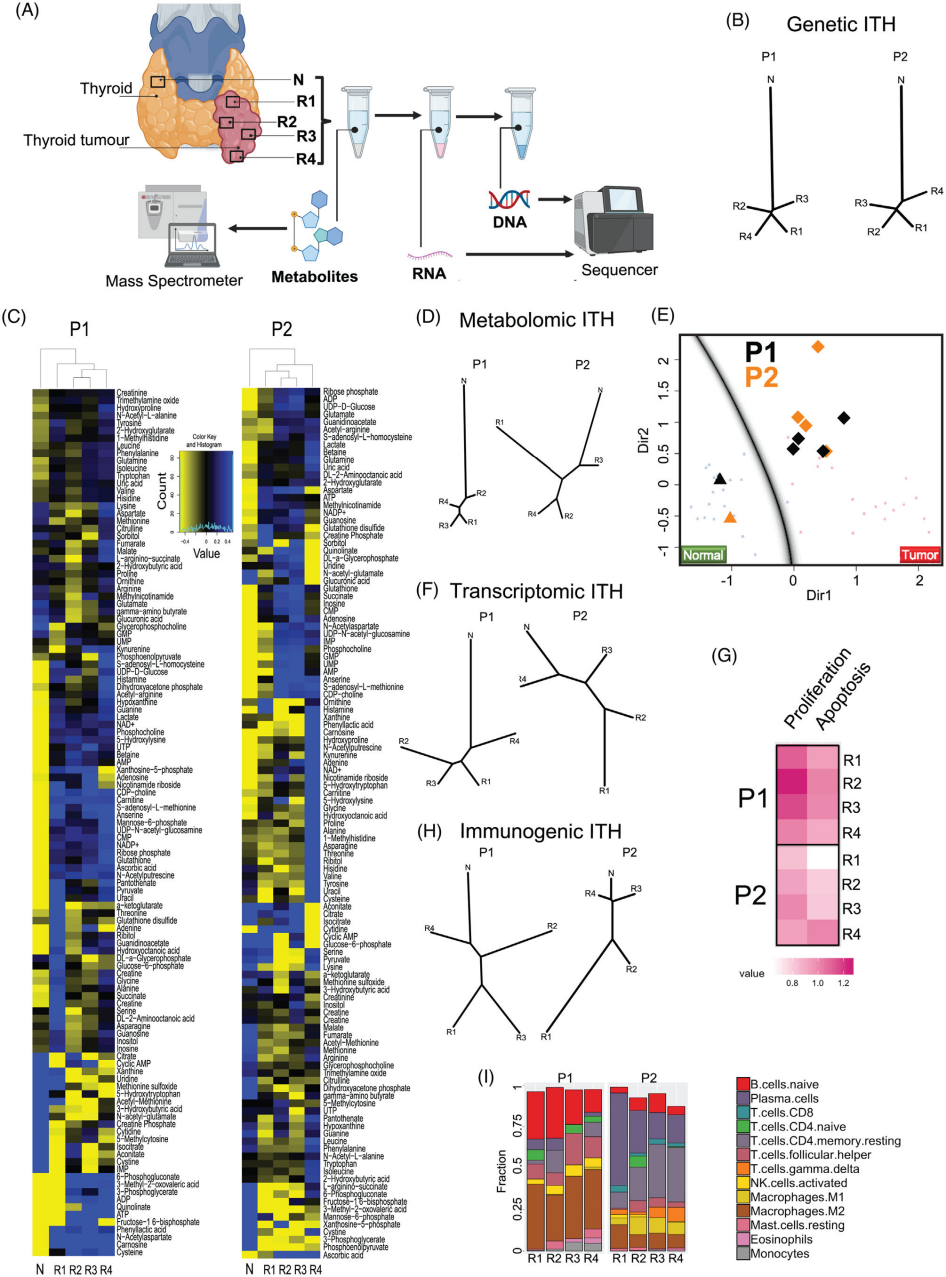
Intra‐tumour heterogeneity (ITH) in papillary thyroid cancer (PTC): (A) step‐by‐step genetic and non‐genetic PTC ITH determinations; (B) dendrogram of genetic ITH from patients 1 (P1) and 2 (P2). N represents normal thyroid tissue, and Rs (R1–R4) represent different pieces of PTC cancer tissues; (C) metabolic heat maps (D) and dendrogram show the distinct metabolic profile in different pieces of cancer in P2; (E) mclust analysis considering the six most significant altered metabolites used to build the model Normal (blue) versus Tumour (red), as shown in Figure 1H. The diamond represents the tumour samples (R1–R4), and the triangles represent the normal samples (N) of P1 (black) and P2 (orange). The shadows of small circles represent the projection of the samples used to develop the classification model; (F) dendrogram of transcriptional ITH; (G) heat map of the proliferative score of cancer samples; (H) dendrogram of immunogenic ITH; (I) compositions of immune cell types infiltrated in cancer tissue.

Intra‐tumour genetic and non‐genetic heterogeneities are established cancer hallmarks,^40^, ^41^ often compromising therapy efficacy.^42^ We examined the extent of genetic and non‐genetic ITHs in two female patients (P1 and P2) of the same age and carrying PTCs large enough for multi‐omics profiling from multiple geographically distinct regions from their primary tumours. We selected four spatially well‐separated areas from each primary tumour to perform metabolomic, whole‐exome sequencing and RNA sequencing of these tumour regions and matched normal thyroid tissues (Figure 5A).

Both patients had low somatic mutation burden and low genetic heterogeneity; most somatic mutations were ubiquitous and detected in all regions within the tumour (Figure 5B), a pattern consistent with other reports.^33^

In contrast, as reported in other tumours, ITH and divergence from the matched normal tissues were considerably higher at the transcriptomic, metabolic and immunogenic levels.^43^ Tumour samples clustered separately from the normal tissues at the metabolite levels (Figure 5C), as observed previously (Figure 1G). However, the metabolic ITH was considerably higher in P2, as shown by principal component analysis (Figure S3A) and dendrograms (Figure 5D). In order to check whether the high metabolic ITH among the tumour samples from P2 could affect the power of our predictive model (Normal vs. Tumour), we combined both data sets (predictive model with ITH samples) and selected the six metabolites we reported as predictive (aconitate, glycine, inosine, isoleucine, proline and taurine) (Figure S3B). To obtain more control over setting up training and testing sets and providing a more accurate predictive model, we also used the support of a vector machine (SVM)^44^ (see Section 4 for details). We found that the sensitivity of this predictive model on the ITH cases using SVM is .7, the specificity is .5 and the area under the ROC curve is .75 (Figure S3C). Using mclust analysis, as expected, all the tumour and normal samples lie in their respective groups despite the high metabolic ITH found in P2 and the small number of samples (Figure 5E). These results suggest that the differences observed among tumour samples were particular to DTC and very distinct from the metabolic profile of normal thyroid tissue, which allowed us to successfully use our predictive model with an external group (ITH samples), thus producing the desired outcome.

The transcriptional ITH was also increased in P2 (Figure 5F). P2 presented a more conspicuous heterogeneous pattern in the proliferative score based on gene expression patterns (Figure 5G). The heterogeneous metabolic and transcriptional profile of P2 reflected its tumour phenotype, which was a mixture of classic papillary type, follicular type, oncocytic features and dense lymphocytic infiltration (7 of 12 metastatic lymph nodes [58%]). On the other hand, P1 presented a non‐metastatic classic type of papillary carcinoma. All the tumour samples from both patients presented very distinct immune cell profiles, which suggests a high immune ITH (Figure 5H). Moreover, there was considerable variation between the patients in their proportional abundance of different immune cell types (Figure 5I), suggesting that the immune microenvironment might differ among PTC samples.

Despite a small sample set, these results support a model that, although genetic variations were minor among the thyroid cancer subtypes, non‐genetic heterogeneity at the metabolic, transcriptional and immune levels within and between tumours, especially in metastatic PTC cases, was substantial.

## 3 DISCUSSION

The potential for overdiagnosis and/or overtreatment in patients with thyroid cancer remains a critical topic for clinical research.^8^, ^9^, ^45^, ^46^ It is clear that DTC requires a more comprehensive procedure to identify patients who should or should not be subjected to more invasive therapies. Another challenge in thyroid cancer translational research is the earlier identification of a potential metastatic PTC, which comprises ∼30% of all cases in the SEER database. Overall, the 10‐year survival of thyroid cancer patients is ∼98%, but those few patients with local and distant metastases are associated with a poor prognosis.^11^, ^47^ Unfortunately, efforts to find a mutational profile that predicts high‐risk PTC, such as ThyroSeq^18^ and Veracyte,^17^ are generally insufficient and may need to be combined with other diagnostic methods.^16^, ^23^ Considering that FNA aligned with cytopathologic analysis may often be inconclusive in many patients, we provide proof‐of‐concept that an integrated analysis of non‐genetic (e.g. metabolomic and cytopathology) and genetic biomarkers (e.g. RNA and/or DNA genome sequencing) has the potential to refine the diagnosis and management of DTC.

The DTC metabolic profile is markedly different from normal thyroid tissue, as we showed, which is sufficient to identify thyroid cancer tissue, even when other conventional methods are inconclusive. Targeted and untargeted studies of thyroid cancer have suggested several potential metabolic signatures.^48^, ^49^, ^50^ Previous reports have proposed using metabolite levels as a potential diagnostic tool.^48^ Most of these metabolites, such as lactate, glucose, glutamine, asparagine and choline, were indeed altered in our metabolic profile.^51^ However, the results presented here showed that other metabolites, such as aconitate, glycine, inosine, isoleucine, proline and taurine, could be more efficient in distinguishing normal thyroid from cancer tissues, which might improve diagnostic accuracy. In addition, we simulated an external cohort using all ITH samples by applying SVM. This test showed that despite the small cohort sizes (Normal vs. Tumour model group and ITH external group), our data result in an efficient tool to be combined with traditional methods and help in DTC diagnosis and management.

Beyond the classification of DTC using the metabolic profile method, it is important to mention that our results suggest an important role of lipid metabolism in DTC samples. Abnormalities in choline and lipid metabolism in human DTC were observed before,^52^ but our data also suggest that alterations in choline levels might happen to supply β‐oxidation with more lipids to obtain energetic balance in tumour cells.

Our study also showed that some metabolite levels were altered in metastatic PTC: adenine, ascorbic acid, betaine, guanidoacetic acid, phenylacetic acid and phenylpyruvate. However, this phenotype must be associated with other cancer features to generate a more reliable distinction between low‐ and high‐risk thyroid cancers. In this particular case, we suggest associating metabolomics with genomic data. Moreover, we might consider more than only BRAF mutations as predictors of poor prognostics.^24^, ^33^, ^53^ In the cancers with BRAF mutations in our cohort, 50% were metastatic, whereas the ones with RET fusions were largely metastatic (80%). Notably, we could not distinguish BRAF mutations and RET fusions at the metabolic level, presumably because they may redundantly trigger the same downstream signal transduction pathway(s). As such, both mutations were mutually exclusive, as shown here, and consistent with reports from other investigators.^54^, ^55^, ^56^ Taken together, this body of work suggests that both of these molecular alterations may ultimately lead to high‐risk PTC generation. However, a major limitation of this discovery work is that our tentative hypothesis‐generating conclusions are based on a relatively small patient cohort. Future studies of patients with PTC presenting with distant metastasis might conceivably shed further light on this currently open question.

Equally important, other alterations must be further investigated as potential high‐risk PTC predictors, such as telomere length, nDNA and mtDNA mutation burden and ITH. Our data show a good correlation between shorter telomere length and cancer risk. Curiously, long telomere length, and not shorter, is associated with multiple cancer.^57^ Generally, the TERT activity is increased in these cancers because shorter telomeres limit the replicative potential of tumour cells and can cause genotoxic stress.^58^ We suggest that further investigation should be conducted to evaluate whether shorter telomere length could represent a specificity of thyroid cancer that might be aggravated by metastatic potential. Again, despite the relatively small patient cohort, we observed a high nDNA and mtDNA mutational burden and ITH in high‐risk PTC when we contrasted multiple primary tumour regions of patients with non‐metastatic versus metastatic PTCs.

We should highlight that (although the methods here were carefully applied) the conclusions of this study have inherited limitations due to its relatively small number of samples and its descriptive nature, which allows us only to suggest that these alterations could favour the identification of a metastatic PTC. Ideally, our results must be validated with a larger patient cohort. Moreover, it is important to mention that we are proposing a method that might complement existing methods and is definitively not meant as a substitute. Finally, the approach shown here should not be limited to DTC. Although not covered in this work, multi‐omics analysis, including a comprehensive metabolic profile, could also be tested to distinguish different thyroid nodules and might be efficient in identifying benign adenomas. This study showed that our classification model could distinguish cancer and normal thyroid tissue. This approach would be beneficial when the cytopathologic report presents indeterminate results and should help the physician to manage the patient, such as deciding whether to submit the patient to surgery, the extension of this surgery and/or radioiodine therapy application.

We also showed that multi‐omics analysis considering telomere length, high nDNA and mtDNA mutation burden and high ITH might help identify high‐risk PTC early. To conclude, we demonstrate that new research avenues in thyroid cancer may still have important implications for the diagnosis, treatment and prognosis of DTC patients.

## 4 METHODS

### 4.1 Thyroid tissue extraction

The tissue was fractioned and classified by a dedicated expert pathologist in loco just after the surgical remotion. Tissues classified as DTC are immediately frozen and stored at −80°C. The DTC samples were picked from four equidistant sites to determine ITH features. Paired normal and tumour thyroid tissue samples were unidentified and maintained by the Rutgers Cancer Institute of New Jersey (CINJ) Biospecimen Repository Service (CINJ‐BRS) under the auspices of IRB‐approved protocol. CINJ‐BRS numbered and linked the stored tissue with its specific surgical report and patient history, removing identification. The samples were obtained from CINJ‐BRS after the protocol above and processed as indicated by the following protocols.

### 4.2 Histology

A sample of each piece of tissue collected was fixed overnight in formalin 10% and then transferred to 70% ethanol for paraffin‐embedded sections. The paraffin blocks were cut and mounted on slides. The slides were deparaffinized, rehydrated and then submitted to haematoxylin–eosin staining.

### 4.3 Tissue pulverization

A total of 25–30 mg of frozen thyroid tissue (duplicates of non‐normal and tumour samples per patient) were weighed and added to a 2 mL round bottom microtube with a −80°C cold yttria Grinding Ball per tube. The tissues were pulverized by using a Retsch CryoMill following three alternating cycles at 5 Hz for 2 min and 25 Hz for 2 min.

### 4.4 Extraction of polar metabolites from human thyroid tissue

To each 2 mL microtube with 25–30 mg of frozen pulverized tissue, a buffer volume equivalent to 20× the sample weight in μL was added. The extraction buffer was 40:40:20 (v/v/v) methanol:acetonitrile:water with .1 M of formic acid. After adding the buffer, the sample was vigorously vortexed and incubated on crushed ice for 10 min. The samples were then vortexed again and centrifuged for 10 min at 16 000*g* at 4°C. The supernatant A was collected and saved, and the pellets were submitted to re‐extraction following the same procedures which generated the supernatant B. Supernatants A and B were mixed and transferred to a clean 1.5 mL microtube with the appropriate volume of 15% NH_4_CO_3_. The samples were stored in a −80°C freezer until analysis by LC–MS.

### 4.5 LC–MS analysis for polar metabolites

The LC–MS method involved hydrophilic interaction liquid chromatography coupled with electrospray ionization to the Q Exactive PLUS Hybrid Quadrupole‐Orbitrap mass spectrometer (Thermo Scientific). The LC separation was performed on an XBridge B.E.H. Amide column (150 mm × 2.1 mm, 2.5 μm particle size, Waters, Milford, MA, USA) by using a gradient of solvent A (95%/5% H_2_O/acetonitrile with 20 mM ammonium acetate and 20 mM ammonium hydroxide, pH 9.4) and solvent B (20%/80% H2O/acetonitrile with 20 mM ammonium acetate and 20 mM ammonium hydroxide, pH 9.4). The gradient was 0 min, 100% B; 3 min, 100% B; 3.2 min, 90% B; 6.2 min, 90% B; 6.5 min, 80% B; 10.5 min, 80% B; 10.7 min, 70% B; 13.5 min, 70% B; 13.7 min, 45% B; 16 min, 45% B; 16.5 min, 100% B; 22 min, 100% B. The flow rate was 300 μL/min. The injection volume was 5 μL, and the column temperature was 25°C. The MS scans were done in positive and negative ionization modes with a mass resolution of 70,000. The automatic gain control target was 3e6. The maximum injection time was 50 ms. The scan range was 75–1000. The metabolite features were extracted in MAVEN^59^ with a mass accuracy window of 5 ppm. The compound identification is based on accurate mass and retention time matching our customized in‐house metabolite library.^60^

### 4.6 DNA and RNA extractions and processing

A total of 20–25 mg of frozen pulverized tissue was submitted to DNA extraction following DNeasy Blood and Tissue Kit (Qiagen ID: 69504) protocol. For the ITH co‐extraction of metabolites, DNA and RNA were extracted using the remaining pellet of tissues from the extraction of the metabolites. After initial quality checks of the raw RNA sequencing reads by using FastQC (v0.11.7) and removal of any low‐quality reads, STAR aligner (v 2.6.0c)^61^ was used to map the remaining reads onto the human genome (GRCh38). RSEM (v1.3.1)^62^ was used for transcript quantification, and log2 (TPM + 1) (transcripts per million) values were reported for different tumour regions and also matched non‐malignant regions. ESTIMATE^63^ was used for predicting tumour purity and the presence of stromal/immune cells in tumour tissues. Multiregional tumour trees at transcriptomic levels were constructed for every patient with RNA expression data for all genes across different regions, an approach similar to that used at the genomic level. Manhattan distance was computed between all regions of a patient sample by using log2 (TPM + 1), and then, unrooted dendrograms were drawn by using this distance metric. Proliferation (PI) and apoptotic (AI) indices were calculated in a similar approach by considering 124 proliferation‐associated genes^64^ and 6 apoptosis‐related genes. For each gene, *z*‐scores corresponding to its expression in tumour regions were calculated based on the mean and standard deviation of its expression in the non‐malignant samples. Then proliferation index (PI) and apoptosis index (AI) were defined as mean *z*‐scores of all proliferation and apoptosis genes, respectively.

### 4.7 Whole‐genome sequencing analysis

Mutect2 from the GATK^65^ is carried out to analyse the point mutations and small indels by considering the tumour‐normal pair mode with the GRCh38 genome. The corresponding results are annotated by SnpEff^65^ and then filtered with a series of conditions: (i) ‘FILTER’ column as ‘PASS’; (ii) tumour sample depth, REF + ALT > 10 and REF + ALT < 200; (iii) normal sample depth, REF + ALT > 8 and REF + ALT < 200; (iv) tumour AF > .2; (v) normal AF < .05; and (vi) SNVs only from 1 to 22, X, Y and MT chromosomes. The mutation signatures are analysed by deconstructSigs^66^ with the filtered SNVs as input. Manta analyses the SVs.^67^ The filter conditions are applied to the original outputs: (i) ‘FILTER’ column as ‘PASS’; (ii) SOMATICSCORE > 40. The copy number variations are investigated by using FACETS.^68^

### 4.8 Mitochondria DNA analysis

Ten tumour‐normal pairs of WGS data were used for somatic mutation calling. First, the sequencing reads were clipped with Trimmomatic (Trimmomatic, RRID:SCR_011848) .39^69^; then, the reads were mapped to reference GRCh38 by using Burrows–Wheeler Alignment tools (bwa .7.17‐r1188).^70^ The bam files (the mapped file format) were further sorted, mark‐duplicated, base quality score recalibrated and indexed using samtools‐1.3.1 5 and gatk‐4.1.9.0. Mitochondria‐mode of Mutect2 (gatk) served to call somatic variants. The variants were filtered by gatk FilterMutercCalls. We developed a complex filter for screening low confident variants by using allele fractions of alternate alleles (AFs) in the tumour (AF > .03) and normal (AF = 0), non‐coding removal, approximate read depth (DP > 2000), and log ten likelihood ratio score of variant existing versus not existing (TLOD > 10). The variants were annotated by using Ensembl Variant Effect Predictor (VARIANT, RRID:SCR_005194; VEP 101.0).^71^ The mtDNA copy number was estimated by the total number of reads mapping to the mitochondrial genome divided by the total number of reads and multiplied by the factor (6 × 10^9/16 × 10^3), which is the size of the diploid genome and the mitochondrial genome.

### 4.9 Telomere length estimation method

We used TelomereHunter^72^ to estimate telomere content from human WGS data considering default settings.^43^ Telomere content = Intra telomeric reads × 10^6^/total reads with telomeric GC.

### 4.10 Somatic mutation calling in ITH samples

FastQC (v0.11.7) was used for initial quality checks, and low‐quality reads and PCR duplicates were removed. Next, we used BWA‐mem^73^ (v0.7.17‐r1188) to map the reads onto the human genome (GRCh38) and call variants by using varScan2^74^ (mapping quality >40, base quality >20). Only ‘high confidence’ somatic variants with tumour allele frequency >5% at least in one tumour region and normal allele frequency <1% were selected. For each somatic variant deemed as a high‐confidence variant in at least one tumour region, we queried the corresponding base position in other tumour regions in that tumour specimen. It was included if reads supported the variant allele with mapping quality >20, base quality >25 and variant allele frequency >2%. All identified somatic mutations were annotated with SnpEff^65^ (v4.3t). Missense, nonsense, frameshift or splicing mutations in known COSMIC cancer genes with high‐predicted impact were marked. The dendrograms were generated with the approach described^43^ in the previous paper from variant allele frequency of variations identified in different regions.

### 4.11 Mutational signatures

Contexts of somatic point mutations were used to draw inferences about their likely etiologies.^75^ We used deconstructSigs^39^, ^66^ to identify patterns of mutational signatures on somatic variants.

### 4.12 Immune cell infiltration

Immune cell infiltrations were inferred from molecular signatures of immune cell types. ESTIMATE (v1.0.13)^63^ was used to predict the immune infiltration level in tumour tissues. CIBERSORT^76^ was used to estimate the abundance of different immune cell populations from expression data. Standard LM22 signature gene file and 1000 permutations were used to calculate deconvolution *p*‐values. Like genomic and transcriptomic data, multiregional tumour trees were made for every patient to infer immunogenic heterogeneity (iITH) with immune cell proportions from CIBERSORT. Manhattan distance was computed between all regions of a patient sample using immune cell proportions data sets independently, and separate unrooted dendrograms were drawn by using respective distance metrics.

### 4.13 Pathways impact analysis and metabolites classification

To evaluate the impact of the metabolic alterations found in tumour versus normal thyroid tissues, we submitted the raw data obtained from the metabolic extraction on MAVEN (ion counts) to log transformation by using the web‐based software MetaboAnalyst 5.0.^77^ The transformed data were used to obtain the class of the metabolites significantly altered and the impact of these alterations on tumour thyroid tissues.^78^

### 4.14 Graphs and figures

All charts and statistics presented in this article were built using GraphPad Prism 9 (GraphPad Prism, RRID:SCR_002798) and RStudio 1.1453. The cartoon in Figure 5A was created with BioRender (BioRender, RRID:SCR_018361). All graphs, pictures and cartoons were assembled by using Adobe Illustrator 26 (Adobe Illustrator, RRID:SCR_010279).

### 4.15 Predictive model and statistical analysis

First, the biomarker levels of the 110 metabolites were transformed by using a log2 transformation. As the number of markers is 0, a small amount, 100, was added to all the biomarkers before taking logs. Thus, the transformation is given by *y* = log2(*x* + 100). As a first step in constructing a predictive model, we used ordinary logistic regression to assess the relationship of each predictor to the sample type. Then the biomarkers that presented *p*‐values smaller than .20/110 = .0018 (Bonferroni adjustment) were selected as predictors unless mentioned differently. The numbers and kinds of predictors are different depending on the comparison. The Lasso regression^25^ was applied to find a subset of biomarkers that effectively predict the status of the sample. Additionally, we used the ‘profL1’ function of the ‘penalized’ R package to select, via cross‐validation, the optimal tuning parameter. Then we used the ‘penalized’ function to conduct the lasso regression.

### 4.16 Support vector machine

We used the “LIBSVM” software^44^ as implemented in the ‘e1071’ R package to build the SVM models (Normal vs. Tumour). As previously described, we combined two data sets into one, selecting the six metabolites we reported as predictive of Normal versus Tumour (aconitate, glycine, inosine, isoleucine, proline, taurine). The ITH data were normalized so that the mean of the normal tissue samples for ITH for each metabolite matches the mean for the corresponding Normal versus Tumour data for normal tissue metabolite. Compared to clustering, SVMs allow more control over setting up training and testing sets and provide more accurate predictive models. We also used cross‐validation with the first data set (with 38 observations) to correct for overfitting by selecting at random 2/3 of the data, building an SVM with that, and testing it on the other 1/3 of the data. We repeat this 1000 times to obtain estimated error rates and confidence intervals for these estimates:

The mean sensitivity is .961, with a 95% confidence interval of .833–1.000.
The mean specificity is .962, with a 95% confidence interval of .800–1.000.
The mean AUC is .998, with a 95% confidence interval of .976–1.000.

Although SVM technology is more accurate for prediction, mclust‐based results are particularly useful for visualization, so we plot from the MclustDR function in the mclust R library, as shown in Figure 5E.

## CONFLICT OF INTEREST STATEMENT

E.W. is a founder of Vescor Therapeutics, has stock in Forma Therapeutics and receives research funding from Deciphera. RP and WA are founders and equity stockholders of PhageNova Bio and MBrace Therapeutics. RP is a paid consultant for PhageNova Bio and serves as its Chief Scientific Officer. RP serves as Chief Scientific Officer and a Board Member, and WA is a Member of the Scientific Advisory Board at MBrace Therapeutics. RP and WA receive research support from PhageNova Bio and MBrace Therapeutics. These arrangements are managed in accordance with the established institutional conflict of interest policy of Rutgers, The State University of New Jersey. Neither PhageNova nor MBrace participated in the present work.

## Supporting information

Supporting Information

Supporting Information

## References

[1] Xing M . Molecular pathogenesis and mechanisms of thyroid cancer. Nat Rev Cancer. 2013;13(3):184‐199. 23429735 10.1038/nrc3431 PMC3791171

[2] Levi F . Cancer incidence in five continents, vol. VI: a review by Fabio Levi. Eur J Cancer. 1993;29(16):2315‐2319.

[3] Morris LGT , Sikora AG , Tosteson TD , Davies L . The increasing incidence of thyroid cancer: the influence of access to care. Thyroid. 2013;23(7):885‐891. 23517343 10.1089/thy.2013.0045 PMC3704124

[4] Siegel RL , Miller KD , Fuchs HE , Jemal A . Cancer statistics, 2021. CA Cancer J Clin. 2021;71(1):7‐33. 33433946 10.3322/caac.21654

[5] Chen AY , Jemal A , Ward EM . Increasing incidence of differentiated thyroid cancer in the United States, 1988–2005. Cancer. 2009;115(16):3801‐3807. 19598221 10.1002/cncr.24416

[6] Kent WDT , Hall SF , Isotalo PA , Houlden RL , George RL , Groome PA . Increased incidence of differentiated thyroid carcinoma and detection of subclinical disease. CMAJ. 2007;177(11):1357‐1361. 18025426 10.1503/cmaj.061730 PMC2072986

[7] Enewold L , Zhu K , Ron E , et al. Rising thyroid cancer incidence in the United States by demographic and tumor characteristics, 1980–2005. Cancer Epidemiol Biomarkers Prev. 2009;18(3):784‐791. 19240234 10.1158/1055-9965.EPI-08-0960 PMC2676561

[8] Haugen BR , Alexander EK , Bible KC , et al. 2015 American Thyroid Association Management Guidelines for adult patients with thyroid nodules and differentiated thyroid cancer: the American Thyroid Association Guidelines Task Force on thyroid nodules and differentiated thyroid cancer. Thyroid. 2016;26(1):1‐133. 26462967 10.1089/thy.2015.0020 PMC4739132

[9] Nabhan F , Ringel MD . Thyroid nodules and cancer management guidelines: comparisons and controversies. Endocr Relat Cancer. 2017;24(2):R13‐R26. 27965276 10.1530/ERC-16-0432 PMC5241202

[10] Nayar R , Ivanovic M . The indeterminate thyroid fine‐needle aspiration: experience from an academic center using terminology similar to that proposed in the 2007 national cancer institute thyroid fine needle aspiration state of the science conference. Cancer Cytopathol. 2009;117(3):195‐202. 10.1002/cncy.20029 19382174

[11] Ho AS , Davies L , Nixon IJ , et al. Increasing diagnosis of subclinical thyroid cancers leads to spurious improvements in survival rates. Cancer. 2015;121(11):1793‐1799. 25712809 10.1002/cncr.29289 PMC4923938

[12] Davies L , Welch HG . Current thyroid cancer trends in the United States. JAMA Otolaryngol Neck Surg. 2014;140(4):317. 10.1001/jamaoto.2014.1 24557566

[13] Bible KC , Kebebew E , Brierley J , et al. 2021 American Thyroid Association Guidelines for management of patients with anaplastic thyroid cancer. Thyroid. 2021;31(3):337‐386. 33728999 10.1089/thy.2020.0944 PMC8349723

[14] Cibas ES , Ali SZ . The 2017 Bethesda system for reporting thyroid cytopathology. Thyroid. 2017;27(11):1341‐1346. 29091573 10.1089/thy.2017.0500

[15] Wang CCC , Friedman L , Kennedy GC , et al. A large multicenter correlation study of thyroid nodule cytopathology and histopathology. Thyroid. 2011;21(3):243‐251. 21190442 10.1089/thy.2010.0243 PMC3698689

[16] Iñiguez‐Ariza NM , Brito JP . Management of low‐risk papillary thyroid cancer. Endocrinol Metab. 2018;33(2):185‐194. 10.3803/EnM.2018.33.2.185 PMC6021317 29947175

[17] Alexander EK , Kennedy GC , Baloch ZW , et al. Preoperative diagnosis of benign thyroid nodules with indeterminate cytology. N Engl J Med. 2012;367(8):705‐715. 22731672 10.1056/NEJMoa1203208

[18] Steward DL , Carty SE , Sippel RS , et al. Performance of a multigene genomic classifier in thyroid nodules with indeterminate cytology: a prospective blinded multicenter study. JAMA Oncol. 2019;5(2):204‐212. 30419129 10.1001/jamaoncol.2018.4616 PMC6439562

[19] Livhits MJ , Zhu CY , Kuo EJ , et al. Effectiveness of molecular testing techniques for diagnosis of indeterminate thyroid nodules: a randomized clinical trial. JAMA Oncol. 2021;7(1):70‐77. 33300952 10.1001/jamaoncol.2020.5935 PMC7729582

[20] Nixon IJ , Simo R , Newbold K , et al. Management of invasive differentiated thyroid cancer. Thyroid. 2016;26(9):1156‐1166. 27480110 10.1089/thy.2016.0064 PMC5118958

[21] Weber T , Klar E . Minimal residual disease in thyroid carcinoma. Semin Surg Oncol. 2001;20(4):272‐277. 11747268 10.1002/ssu.1044

[22] Brito JP , Ito Y , Miyauchi A , Tuttle RM . A clinical framework to facilitate risk stratification when considering an active surveillance alternative to immediate biopsy and surgery in papillary microcarcinoma. Thyroid. 2016;26(1):144‐149. 26414743 10.1089/thy.2015.0178 PMC4842944

[23] Janjua N , Wreesmann VB . Aggressive differentiated thyroid cancer. Eur J Surg Oncol. 2018;44(3):367‐377. 29169931 10.1016/j.ejso.2017.09.019

[24] D'Cruz AK , Vaish R , Vaidya A , et al. Molecular markers in well‐differentiated thyroid cancer. Eur Arch Oto‐Rhino‐Laryngol. 2018;275(6):1375‐1384. 10.1007/s00405-018-4944-1 29626249

[25] Hastie T , Tibshirani R , Friedman J . The elements of statistical learning. Springer Ser Stat. 2009;26(4):505‐516.

[26] Scrucca L , Fop M , Murphy TB , Raftery AE . mclust 5: clustering, classification and density estimation using. R J. 2016;8(1):289‐317. 27818791 PMC5096736

[27] Fraley C , Raftery AE . MCLUST: software for model‐based cluster analysis. J Classif. 1999;16(2):297‐306.

[28] Stephens FB , Constantin‐teodosiu D , Greenhaff PL . New insights concerning the role of carnitine in the regulation of fuel metabolism in skeletal muscle. J Physiol. 2007;581(2):431‐444. 17331998 10.1113/jphysiol.2006.125799 PMC2075186

[29] Cararo‐Lopes E , Dias MH , da Silva MS , et al. Autophagy buffers Ras‐induced genotoxic stress enabling malignant transformation in keratinocytes primed by human papillomavirus. Cell Death Dis. 2021;12(2):194. doi:10.1038/s41419-021-03476-3 33602932 PMC7892846

[30] Cabanillas ME , McFadden DG , Durante C . Thyroid cancer. Lancet. 2016;388(10061):2783‐2795. 27240885 10.1016/S0140-6736(16)30172-6

[31] Cohen Y , Xing M , Mambo E , et al. BRAF mutation in papillary thyroid carcinoma. J Natl Cancer Inst. 2003;95(8):625‐627. 12697856 10.1093/jnci/95.8.625

[32] Santoro M , Carlomagno F . Central role of RET in thyroid cancer. Cold Spring Harb Perspect Biol. 2013;5(12):a009233. 24296167 10.1101/cshperspect.a009233 PMC3839608

[33] Agrawal N , Akbani R , Aksoy BA , et al. Integrated genomic characterization of papillary thyroid carcinoma. Cell. 2014;159(3):676‐690. 25417114 10.1016/j.cell.2014.09.050 PMC4243044

[34] Chatterjee A , Mambo E , Sidransky D . Mitochondrial DNA mutations in human cancer. Oncogene. 2006;25(34):4663‐4674. 16892080 10.1038/sj.onc.1209604

[35] Yuan Y , Ju YS , Kim Y , et al. Comprehensive Molecular Characterization of Mitochondrial Genomes in Human Cancers [published online ahead of print: 2017]. bioRxiv. doi:10.1101/161356

[36] Alexandrov LB , Kim J , Haradhvala NJ , et al. The repertoire of mutational signatures in human cancer. Nature. 2020;578(7793):94‐101. 32025018 10.1038/s41586-020-1943-3 PMC7054213

[37] Ron E , Lubin JH , Shore RE , et al. Thyroid cancer after exposure to external radiation: a pooled analysis of seven studies. Radiat Res. 1995;141(3):259‐277. 7871153

[38] Pleguezuelos‐Manzano C , Puschhof J , Rosendahl Huber A , et al. Mutational signature in colorectal cancer caused by genotoxic pks + *E. coli* . Nature. 2020;580(7802):269‐273. 32106218 10.1038/s41586-020-2080-8 PMC8142898

[39] Alexandrov LB , Nik‐Zainal S , Wedge DC , et al. Signatures of mutational processes in human cancer. Nature. 2013;500(7463):415‐421. 23945592 10.1038/nature12477 PMC3776390

[40] Biswas A , De S . Drivers of dynamic intratumor heterogeneity and phenotypic plasticity. Am J Physiol – Cell Physiol. 2021;320(5):C750‐C760. 33657326 10.1152/ajpcell.00575.2020 PMC8163571

[41] Hanahan D . Hallmarks of cancer: new dimensions. Cancer Discov. 2022;12(1):31‐46. 35022204 10.1158/2159-8290.CD-21-1059

[42] McGranahan N , Swanton C . Biological and therapeutic impact of intratumor heterogeneity in cancer evolution. Cancer Cell. 2015;27(1):15‐26. 25584892 10.1016/j.ccell.2014.12.001

[43] Sharma A , Merritt E , Hu X , et al. Non‐genetic intra‐tumor heterogeneity is a major predictor of phenotypic heterogeneity and ongoing evolutionary dynamics in lung tumors. Cell Rep. 2019;29(8):2164‐2174.e5. 31747591 10.1016/j.celrep.2019.10.045 PMC6952742

[44] Chang C‐C , Lin C‐J . LIBSVM: a library for support vector machines. ACM Trans Intell Syst Technol. 2011;2:1‐27.

[45] Brito JP , Gionfriddo M , Morris JC , Montori VM . Overdiagnosis of thyroid cancer and Graves’ disease. Thyroid. 2014;24(2):402‐403. 23931639 10.1089/thy.2013.0425

[46] Lim H , Devesa SS , Sosa JA , Check D , Kitahara CM . Trends in thyroid cancer incidence and mortality in the United States, 1974–2013. JAMA – J Am Med Assoc. 2017;317(13):1338‐1348. 10.1001/jama.2017.2719 PMC8216772 28362912

[47] Yang L , Shen W , Sakamoto N . Population‐based study evaluating and predicting the probability of death resulting from thyroid cancer and other causes among patients with thyroid cancer. J Clin Oncol. 2013;31(4):468‐474. 23270002 10.1200/JCO.2012.42.4457

[48] Khatami F , Payab M , Sarvari M , et al. Oncometabolites as biomarkers in thyroid cancer: a systematic review. Cancer Manag Res. 2019;11:1829‐1841. 30881111 10.2147/CMAR.S188661 PMC6395057

[49] Yao Z , Yin P , Su D , et al. Serum metabolic profiling and features of papillary thyroid carcinoma and nodular goiter. Mol Biosyst. 2011;7(9):2608‐2614. 21713270 10.1039/c1mb05029j

[50] Xu Y , Zheng X , Qiu Y , Jia W , Wang J , Yin S . Distinct metabolomic profiles of papillary thyroid carcinoma and benign thyroid adenoma. J Proteome Res. 2015;14(8):3315‐3321. 26130307 10.1021/acs.jproteome.5b00351

[51] Wishart DS , Mandal R , Stanislaus A , Ramirez‐Gaona M . Cancer metabolomics and the human metabolome database. Metabolites. 2016;6(1):10. 26950159 10.3390/metabo6010010 PMC4812339

[52] Li Y , Chen M , Liu C , et al. Metabolic changes associated with papillary thyroid carcinoma: a nuclear magnetic resonance‐based metabolomics study. Int J Mol Med. 2018;41(5):3006‐3014. 29484373 10.3892/ijmm.2018.3494

[53] Nikiforov YE , Ohori NP , Hodak SP , et al. Impact of mutational testing on the diagnosis and management of patients with cytologically indeterminate thyroid nodules: a prospective analysis of 1056 FNA samples. J Clin Endocrinol Metab. 2011;96(11):3390‐3397. 21880806 10.1210/jc.2011-1469 PMC3205883

[54] Stransky N , Cerami E , Schalm S , et al. The landscape of kinase fusions in cancer. Nat Commun. 2014;5(1), 4846. doi:10.1038/ncomms5846 25204415 PMC4175590

[55] Yakushina VD , Lerner LV , Lavrov AV . Gene fusions in thyroid cancer. Thyroid. 2018;28(2):158‐167. 29281951 10.1089/thy.2017.0318

[56] Cantwell‐Dorris ER , O'Leary JJ , Sheils OM . BRAFV600E: implications for carcinogenesis and molecular therapy. Mol Cancer Ther. 2011;10(3):385‐394. 21388974 10.1158/1535-7163.MCT-10-0799

[57] McNally EJ , Luncsford PJ , Armanios M . Long telomeres and cancer risk: the price of cellular immortality. J Clin Invest. 2019;129(9):3474. 31380804 10.1172/JCI120851 PMC6715353

[58] Wong K , Robles‐Espinoza CD , Rodriguez D , et al. Association of the POT1 germline missense variant p.I78T with familial melanoma. JAMA Dermatol. 2019;155(5):604‐609. 30586141 10.1001/jamadermatol.2018.3662 PMC6506889

[59] Melamud E , Vastag L , Rabinowitz JD . Metabolomic analysis and visualization engine for LC–MS data. Anal Chem. 2010;82(23):9818‐9826. 21049934 10.1021/ac1021166 PMC5748896

[60] Lu W , Su X , Klein MS , Lewis IA , Fiehn O , Rabinowitz JD . Metabolite measurement: pitfalls to avoid and practices to follow. Annu Rev Biochem. 2017;86:277. 28654323 10.1146/annurev-biochem-061516-044952 PMC5734093

[61] Dobin A , Davis CA , Schlesinger F , et al. STAR: ultrafast universal RNA‐seq aligner. Bioinformatics. 2013;29(1):15‐21. 23104886 10.1093/bioinformatics/bts635 PMC3530905

[62] Li B , Dewey CN . RSEM: accurate transcript quantification from RNA‐Seq data with or without a reference genome. BMC Bioinformatics. 2011;12(1):1‐16. 21816040 10.1186/1471-2105-12-323 PMC3163565

[63] Yoshihara K , Shahmoradgoli M , Martínez E , et al. Inferring tumour purity and stromal and immune cell admixture from expression data. Nat Commun. 2013;4(1):1‐11. 10.1038/ncomms3612 PMC3826632 24113773

[64] Chen Z , Cheng K , Walton Z , et al. A murine lung cancer co‐clinical trial identifies genetic modifiers of therapeutic response. Nat. 2012;483(7391):613‐617. 10.1038/nature10937 PMC3385933 22425996

[65] Cingolani P , Platts A , Wang LL , et al. A program for annotating and predicting the effects of single nucleotide polymorphisms, SnpEff: SNPs in the genome of *Drosophila melanogaster* strain w1118; iso‐2; iso‐3. Fly (Austin). 2012;6(2):80. 22728672 10.4161/fly.19695 PMC3679285

[66] Rosenthal R , McGranahan N , Herrero J , Taylor BS , Swanton C . deconstructSigs: delineating mutational processes in single tumors distinguishes DNA repair deficiencies and patterns of carcinoma evolution. Genome Biol. 2016;17(1):1‐11. 26899170 10.1186/s13059-016-0893-4 PMC4762164

[67] Chen X , Schulz‐Trieglaff O , Shaw R , et al. Manta: rapid detection of structural variants and indels for germline and cancer sequencing applications. Bioinformatics. 2016;32(8):1220‐1222. 26647377 10.1093/bioinformatics/btv710

[68] Shen R , Seshan VE . FACETS: allele‐specific copy number and clonal heterogeneity analysis tool for high‐throughput DNA sequencing. Nucleic Acids Res. 2016;44(16):1‐9. 27270079 10.1093/nar/gkw520 PMC5027494

[69] Bolger AM , Lohse M , Usadel B . Trimmomatic: a flexible trimmer for Illumina sequence data. Bioinformatics. 2014;30(15):2114‐2120. 24695404 10.1093/bioinformatics/btu170 PMC4103590

[70] Li H . Aligning Sequence Reads, Clone Sequences and Assembly Contigs with BWA‐MEM [Published Online Ahead of Print: March 16, 2013]. Cited January 11, 2022. https://arxiv.org/abs/1303.3997v2

[71] Howe KL , Achuthan P , Allen J , et al. Ensembl 2021. Nucleic Acids Res. 2021;49(D1):D884‐D891. 33137190 10.1093/nar/gkaa942 PMC7778975

[72] Feuerbach L , Sieverling L , Deeg KI , et al. TelomereHunter – in silico estimation of telomere content and composition from cancer genomes. BMC Bioinformatics. 2019;20(1):1‐11. 31138115 10.1186/s12859-019-2851-0 PMC6540518

[73] Li H , Durbin R . Fast and accurate short read alignment with Burrows‐Wheeler transform. Bioinformatics. 2009;25(14):1754‐1760. 19451168 10.1093/bioinformatics/btp324 PMC2705234

[74] Koboldt DC , Zhang Q , Larson DE , et al. VarScan 2: somatic mutation and copy number alteration discovery in cancer by exome sequencing. Genome Res. 2012;22(3):568‐576. 22300766 10.1101/gr.129684.111 PMC3290792

[75] Hu X , Xu Z , De S . Characteristics of mutational signatures of unknown etiology. NAR cancer. 2020;2(3):zcaa026. doi:10.1093/NARCAN/ZCAA026 33015626 PMC7520824

[76] Gentles AJ , Newman AM , Liu CL , et al. The prognostic landscape of genes and infiltrating immune cells across human cancers. Nat Med. 2015;21(8):938‐945. 26193342 10.1038/nm.3909 PMC4852857

[77] Chong J , Wishart DS , Xia J . Using MetaboAnalyst 4.0 for comprehensive and integrative metabolomics data analysis. Curr Protoc Bioinforma. 2019;68(1):e86. 10.1002/cpbi.86 31756036

[78] Xia J , Wishart DS . Web‐based inference of biological patterns, functions and pathways from metabolomic data using MetaboAnalyst. Nat Protoc. 2011;6(6):743‐760. 21637195 10.1038/nprot.2011.319

