# Supplementary material for: Integrated metabolic and genetic analysis reveals distinct features of human differentiated thyroid cancer
Source: Clin Transl Med. 2023 Jun 14;13(6):e1298. doi: 10.1002/ctm2.1298 (PMC10267429; doi:10.1002/ctm2.1298)
Supplement: Supplementary file 2 — Supporting Information [file CTM2-13-e1298-s002.docx]

**SUPPLEMENTARY FIGURES AND TABLES**

**
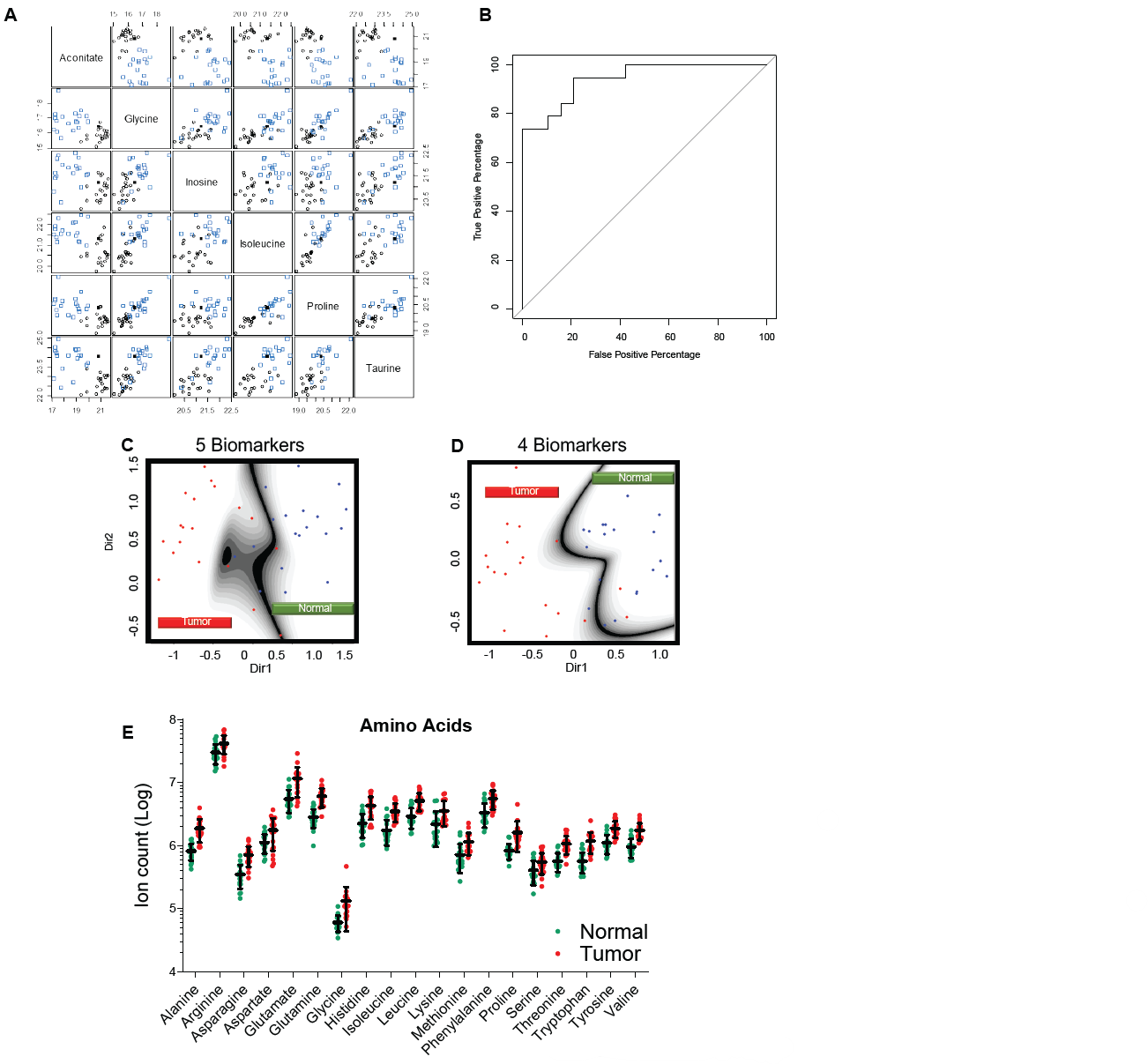
**

**Supplementary Figure S1:** (A) Matrix of scatterplots of each of the six biomarkers versus the others, with "blue squares" indicating cancer samples and "black circles" indicating normal samples. (B) The receiver operating characteristic curve (ROC) shows the power of the predictive model. mClust analysis considering (C) five (glutamate, leucine, proline, taurine, and threonine) and (D) four (glutamate, proline, taurine, and threonine) most altered metabolites. The cross-validated and standard error rates are 0.1579 - 0.055 for five biomarkers and 0.236 - 0.059 for four biomarkers. (E) Levels of proteogenic amino acids found in samples of DTC patients.


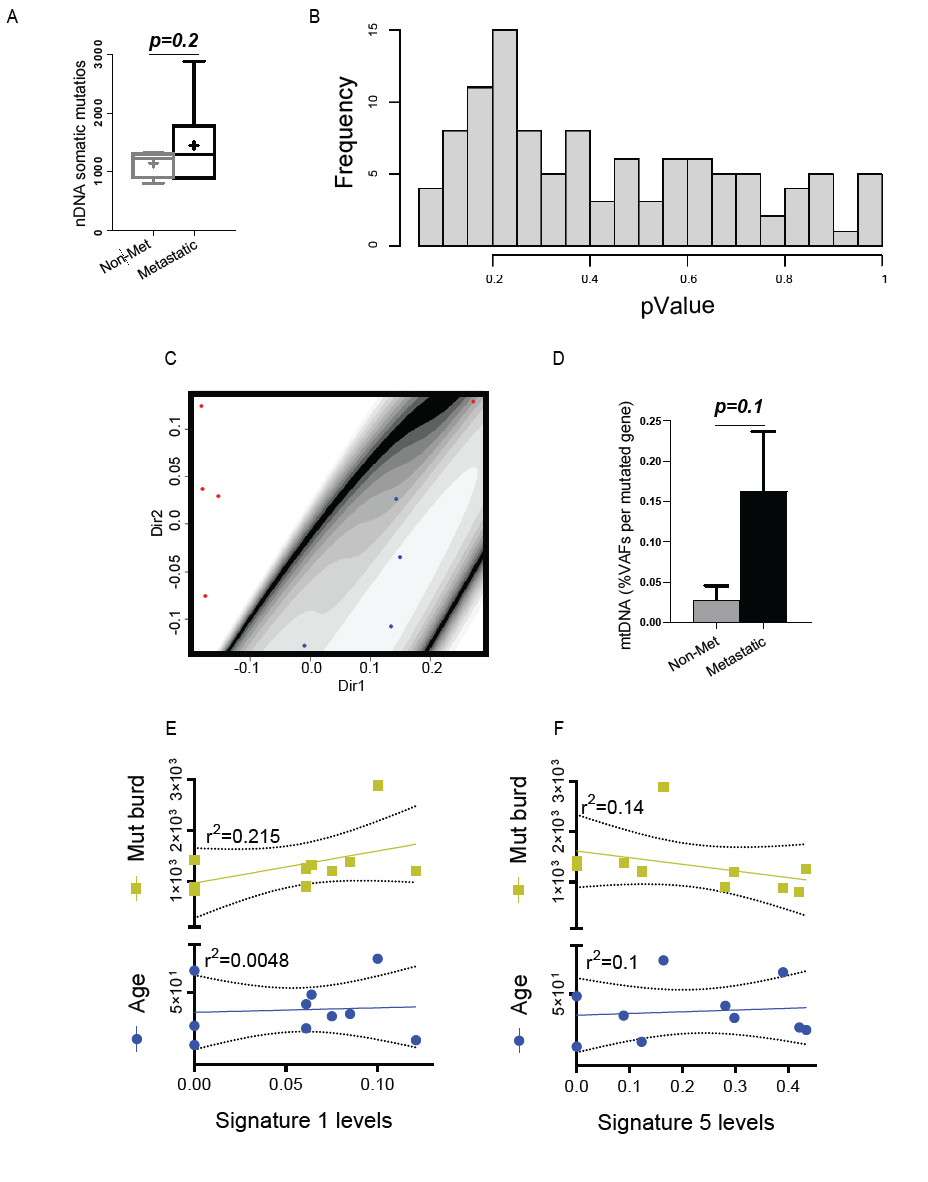


**Supplementary Figure S2:** (A) Nuclear DNA mutation burden in metastatic and non-metastatic PTC. (B) Logistic regression analysis of BRAF mutation versus CCDC6-RET fusion comparison presenting the *P*-values per sample. The smallest p-value in this comparison was 0.0760 (thiamine), emphasizing no correlation between the metabolic profile and genetic alterations. (C) mClust analysis considering the six main altered metabolites comparing the groups in B. (D) mtDNA mutation burden in metastatic and non-metastatic PTC. (E) and (F) Correlation of nDNA mutation burden and age with mutational signatures 1 and 5, respectively. Data presented as mean (SEM) *t*-test.

**
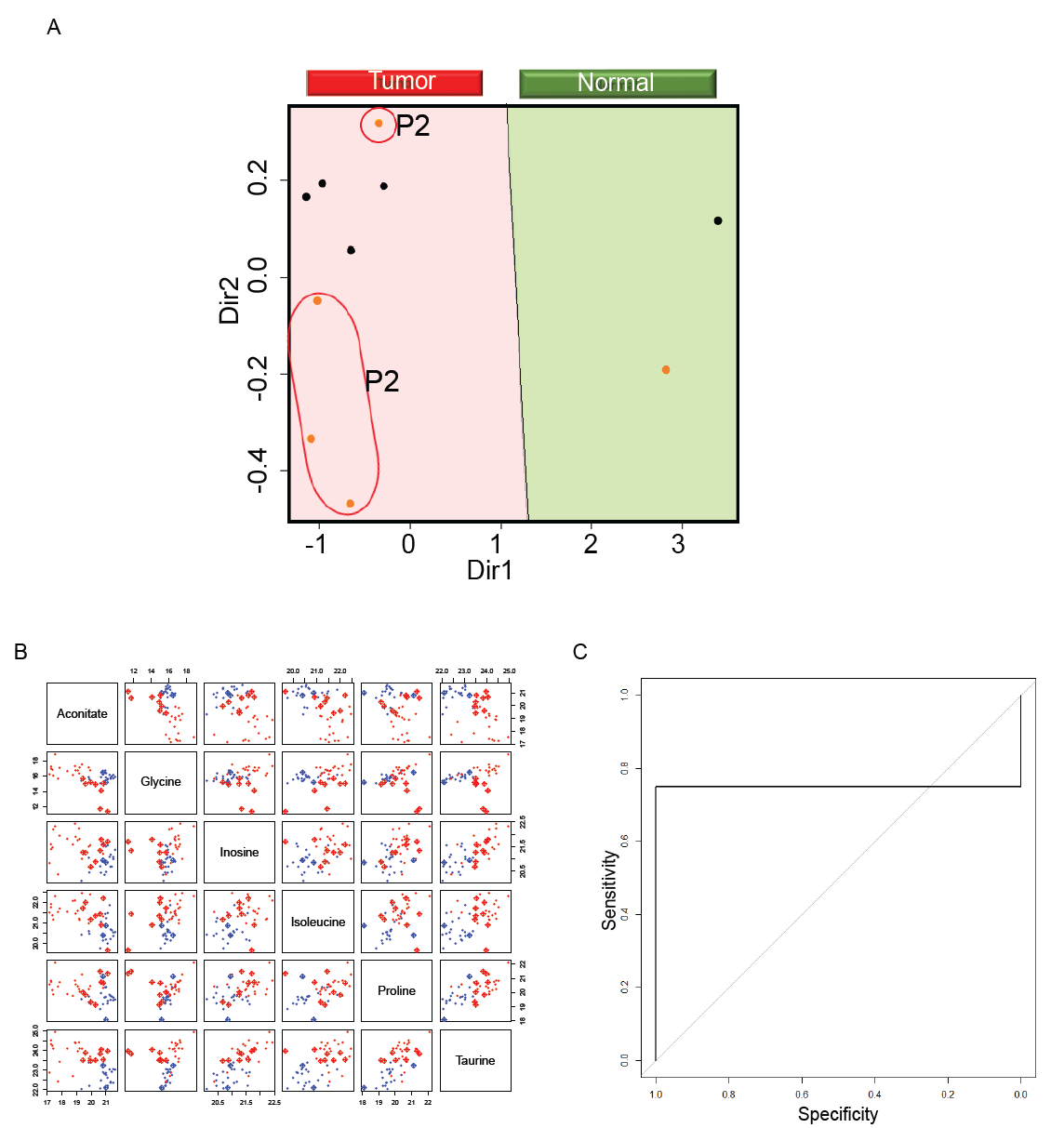
Supplementary Figure S3:** (A) Principal component analysis considering only the ITH samples. (B) Matrix of scatterplots of each of the six biomarkers versus the others. The cancer samples are in red, while the normal samples are in blue. The diamond represents the ITH samples. The circles represent the samples used to build the classification model Normal vs. Tumor. The receiver operating characteristic curve (ROC) shows the power of the predictive Normal vs. Tumor model in ITH samples.

**
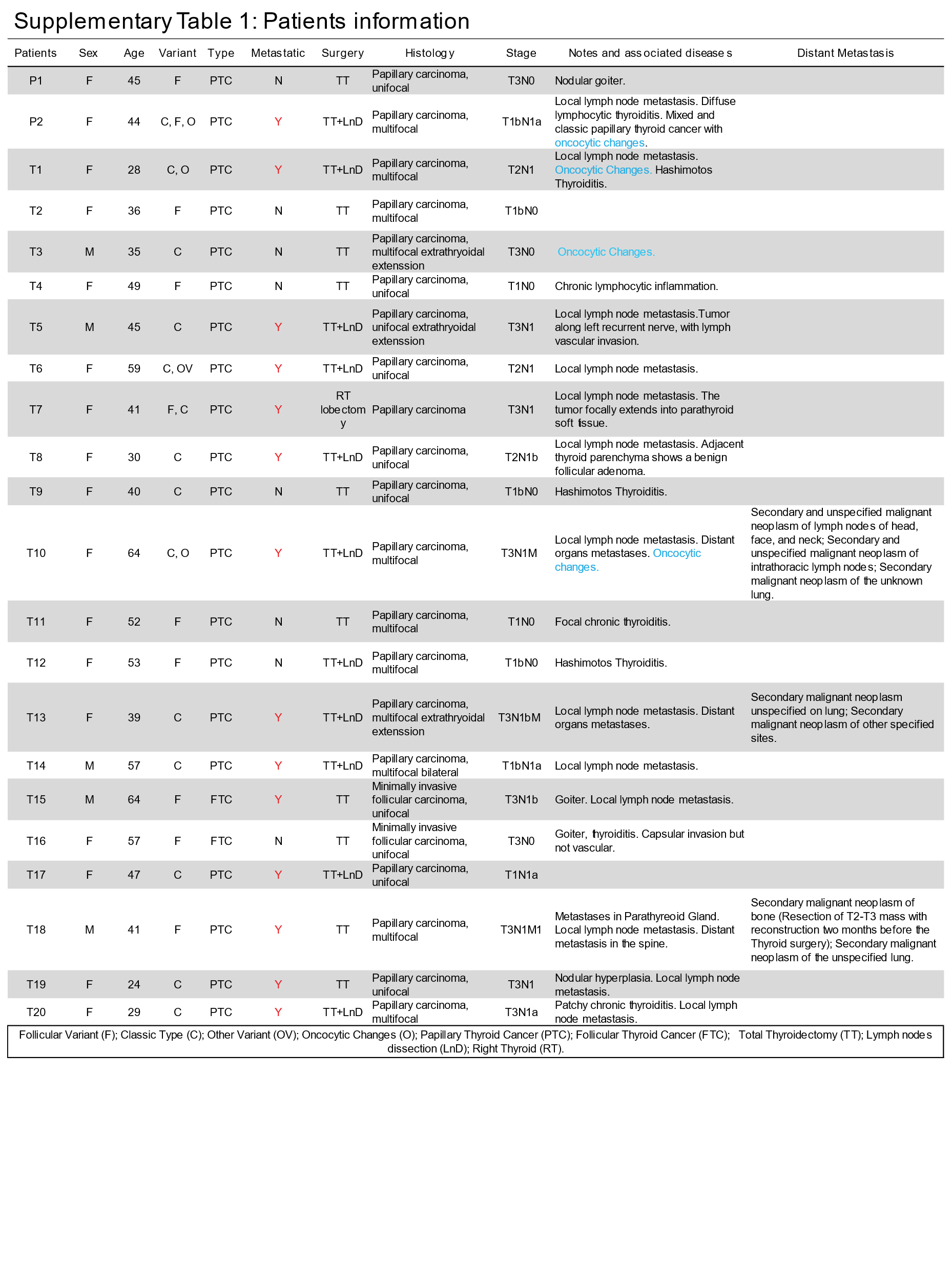
**

**
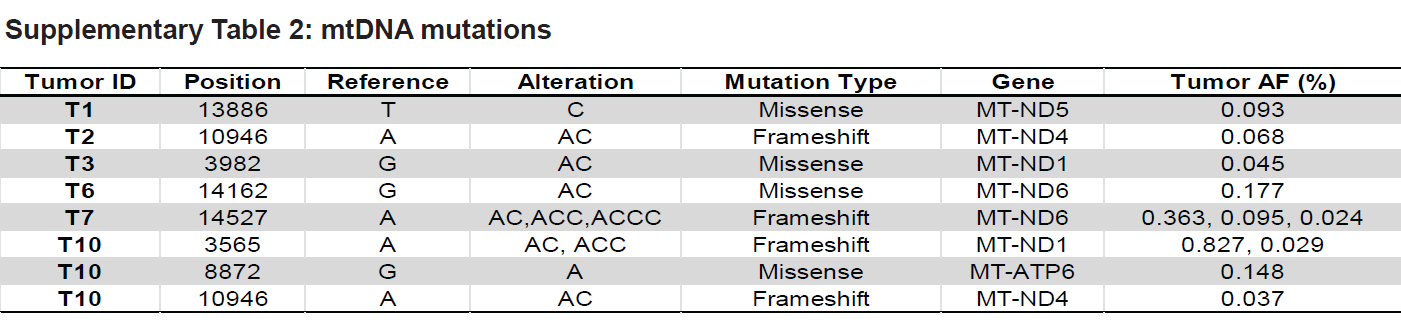
**
